# A Mobile NMR Sensor and Relaxometric Method to Non-destructively Monitor Water and Dry Matter Content in Plants

**DOI:** 10.3389/fpls.2021.617768

**Published:** 2021-02-05

**Authors:** Carel W. Windt, Moritz Nabel, Johannes Kochs, Siegfried Jahnke, Ulrich Schurr

**Affiliations:** ^1^Institute for Bio- and Geosciences IBG-2: Plant Sciences, Forschungszentrum Jülich, Jülich, Germany; ^2^Faculty of Biology Biodiversity, University of Duisburg-Essen, Essen, Germany

**Keywords:** NMR, time domain, sensor, dry matter, water content, source-sink, leaf water potential, pressure–volume curve

## Abstract

Water content (WC) and dry matter content (DMC) are some of the most basic parameters to describe plant growth and yield, but are exceptionally difficult to measure non-invasively. Nuclear Magnetic Resonance (NMR) relaxometry may fill this methodological gap. It allows non-invasive detection of protons in liquids and solids, and on the basis of these measures, can be used to quantify liquid and dry matter contents of seeds and plants. Unfortunately, most existing NMR relaxometers are large, unwieldy and not suitable to measure intact plants or to be used under field conditions. In addition, currently the appropriate NMR relaxometric methods are poorly suited for non-expert use. We here present a novel approach to overcome these drawbacks. We demonstrate that a basic NMR relaxometer with the capability to accept intact plants, in combination with straightforward NMR and data processing methods, can be used as an NMR plant sensor to continuously, quantitatively and non-invasively monitor changes in WC and DMC. This can be done *in vivo, in situ*, and with high temporal resolution. The method is validated by showing that measured liquid and solid proton densities accurately reflect WC and DMC of reference samples. The NMR plant sensor is demonstrated in an experimental context by monitoring WC of rice leaves under osmotic stress, and by measuring the dynamics of water and dry matter accumulation during seed filling in a developing wheat ear. It is further demonstrated how the method can be used to estimate leaf water potential on the basis of changes in leaf water content.

## Introduction

Dry weight (DW), fresh weight (FW), dry matter content (DMC), and water content (WC) are some of the most central parameters to describe plant growth and water status. However, they usually are determined gravimetrically, which means, destructively. This makes it extremely difficult to measure or monitor the dynamics of these traits in the living plant. To circumvent this problem a variety of sensor technologies approximate FW and WC by correlation with other parameters that are more readily measured in the living plant. However, currently no method allows to non-invasively measure DW or DMC directly.

The oldest and most intuitive methods to compare growth of plants involve measuring weight or size. Weighing easily yields information on FW and DW, but requires harvesting of plants or plant parts. Size in terms of length, surface area, or volume can be measured either manually, or in high throughput with the help of computer vision (Scharr et al., 2017). Some of the earliest electronic sensors to monitor growth are transducers (dendrometers) that measure growth or shrinkage of stems, fruits and leaves (Drew and Downes, 2009; Fernández and Cuevas, 2010; Seelig et al., 2012; Afzal et al., 2017). Diameter and thickness variations contain a wealth of information regarding growth, reversible expansion and contraction, but are also used to monitor plant water status in response to abiotic stresses (Fernández and Cuevas, 2010; Seelig et al., 2012). However, even though diameter variations and secondary growth will be correlated to changes in FW and DW, dendrometers cannot measure them directly.

Most of the recently developed sensors that detect changes in FW or WC exploit the electromagnetic properties of plant tissue, by measuring absorption or reflection of electromagnetic radiation, changes in the dielectric constant, or both. A number of examples are terahertz time-domain spectroscopy (Born et al., 2014; Santesteban et al., 2015), microwave resonance (Dadshani et al., 2015; Sydoruk et al., 2016), or thermal sensing (Atherton et al., 2012). In the radio frequency (rf) range, methods such as time domain reflectometry (TDR) (Constantz and Murphy, 1990), frequency domain capacitance (FD) (Holbrook et al., 1992), and fringing field FD (Zhou et al., 2015, 2018) have found application. Another method is frequency domain reflectometry, which is used in trees to measure volumetric WC, but requires holes to be drilled in the trunks (Hao et al., 2013; Zhou et al., 2015). These methods are fast, affordable and well suited for sensors, but are not specific in what they measure. The absorption of radiation and changes in the dielectric constant of a plant will correlate with changes in plant WC, but also with other parameters such as changes in ionic or solute content. Quantitative interpretation of such sensor data therefore remains challenging (Fiorani et al., 2012).

Nuclear Magnetic Resonance (NMR) is a more specific sensing technology. It is best known for its applications in Nuclear Magnetic Resonance Imaging (MRI, also abbreviated to MR imaging, or NMRi) in human medicine, but also has found many applications in plant science. Recent applications of MRI include imaging plant anatomy, for example of tree stems affected by disease (Kuroda et al., 2006), bud ontogeny (Hesse et al., 2018), imaging of fruits and crops (Glidewell, 2006), water content and cavitation events in stems (Holbrook et al., 2001; Choat et al., 2010; De Schepper et al., 2012; Hochberg et al., 2016; Van de Wal et al., 2017; Meixner et al., 2020), structure and function of roots in soils (Jahnke et al., 2009; Kaufmann et al., 2009; van Dusschoten et al., 2016), imaging xylem- and phloem sap flow in stems and petioles (Scheenen et al., 2000, 2007; Windt et al., 2006, 2009), or micro imaging of seeds (Sreenivasulu et al., 2010; Munz et al., 2017). For comprehensive overviews we refer to Van As (2007) and Borisjuk et al. (2012).

NMR utilizes the fact that protons, as found in every water and organic molecule, possess a magnetic moment. Inside a strong magnetic field protons will therefore orient themselves along the direction of the field and process about it with a frequency (Larmor frequency) that scales linearly with the magnetic field strength. These spinning protons (spins) can be brought out of equilibrium by a radio pulse with this specific resonance frequency. In the rf coil of an NMR scanner these excited spins will induce an rf signal, the amplitude of which is directly proportional to the number of spins in the sample. After excitation the NMR signal of the spins relaxes exponentially, with a time constant that is characteristic for their physicochemical environment (Van As, 2007). Two types of signal relaxation can be distinguished, spin-lattice relaxation (T_1_) and spin-spin relaxation (T_2_). T_1_ relaxation occurs as a result of the transfer of energy from spins to surrounding molecules (the lattice). For our purposes T_2_ is the most relevant relaxation mechanism. It is faster and more straightforward to measure and is much more sensitive to changes in proton mobility than T_1_. It is the latter property of T_2_ that in the current study is exploited to make a distinction between solid and liquid proton bearing matter. Protons in molecules that can freely move about (e.g., in pure liquid water) have very long T_2_ relaxation times with values of up to 2 s. If the solution is more viscous, or water is enclosed in small spaces, T_2_ will be shorter. In oils, T_2_ relaxation times in the tens of milliseconds can be expected. At the lowest extreme of the mobility spectrum, protons in solids exhibit T_2_ values that are shorter still, with values in the microsecond range. Based on these relaxation differences, time-domain NMR (TD-NMR) can quantitatively distinguish signal of protons in solids, from that of protons in liquids.

TD-NMR is a well-established method to characterize properties of materials and foods (van Duynhoven et al., 2010). The utility of TD-NMR for the plant sciences has also been well recognized (Borisjuk et al., 2012; Musse et al., 2013; Van As and van Duynhoven, 2013). In fact, some of the earliest applications of (steady-state) NMR involved the estimation of water content in starch suspensions and plant tissues (Shaw and Elsken, 1956) and an early example of TD-NMR was the determination of WC in wood (Sharp et al., 1976). Compared to scanners for NMR imaging (MRI), equipment for TD-NMR is somewhat more affordable and modest in size. Still, due to the restrictive geometries of NMR magnets and coils of most commercial relaxometers, the use of TD-NMR has so far been mostly limited to destructive and lab-based analyses. A notable exception is NMR in the petrochemical industry, where rugged inside-out NMR oil well logging tools are used to measure water and oil contents of surrounding rock (Ellis and Singer, 2007).

Two bottlenecks currently limit the application of TD-NMR in the plant sciences. The first is the lack of suitable hardware, i.e., small-scale NMR magnets that allow access to intact plants, and mobile spectrometers that can be used in the greenhouse or field. To address this problem several groups developed their own hardware and methods, each striking a different balance between mobility and performance. The first to bring an NMR system capable of TD-NMR into the greenhouse were Van As et al. (1994), in a system that was used to measure xylem sap flow in plants. Geya et al. (2013) moved a full-size NMR scanner (100 kg spectrometer plus 60 kg magnet) into a pear orchard to measure fruit development by TD-NMR, but due to the size and weight of the device required an electric trolley to move it there. Malone et al. (2016) employed resistive, super low-field NMR magnets to measure stem WC in a tree in a greenhouse. Their system was very light in weight, but because of the low field strength suffered from an extremely low signal to noise ratio. Capitani et al. (2009) used a small unilateral NMR device to measure relative leaf WC under drought. Musse et al. (2017) took a different approach by making the entire NMR lab mobile. They put a conventional TD-NMR scanner in a minivan and parked it next to a field to assess the water status and sub-cellular distribution of cut out leaf samples. Our laboratory, finally, previously demonstrated the use of a small scale 3.5 kg Halbach magnet, roughly the size of a soda can, to measure diurnal variations in stem WC in poplar (Windt et al., 2011). We subsequently developed simpler, more robust C-shaped magnets and applied them to monitor WC of growing pods in bean (Windt and Blümler, 2015) and changes in the WC of stems and succulent leaves of mangrove saplings exposed to varying soil salinities (Lechthaler et al., 2016). A modified version of these C-shaped magnets, fitted with improved tuning electronics, is at the basis of the NMR sensor utilized in this work.

The second bottleneck is that TD-NMR of plants usually involves complicated procedures and data processing methods. For simple, homogeneous samples (e.g., tube of water) this would not be the case. If all protons experience the same physicochemical environment and therefore exhibit the same relaxation behavior, one could fit the T_2_ decay of the NMR signal with a single mono-exponential function:

(1) $$A=A_{0}e^{-t/T_{2}}$$

The proton density (A_0_) then equals the Y intercept of the fitted curve and is easily determined (Van As, 2007). If, however, in a sample many proton fractions with different physicochemical environments exist, the decay curve will become a summation of the curves of all component fractions. Intact plant organs contain many tissue types and cells of all shapes and sizes, thus giving rise to a multitude of proton fractions with larger or smaller differences in T_2_ relaxation times. If the number of fractions in a sample is known, are well separated in terms of T_2_, and can be approximated with 4 or fewer exponentials, then a multi-exponential fit may be used to fit and quantitatively resolve the proton density of the different fractions (Musse et al., 2010). This and similar techniques, however, require either well described samples, or expert operators to yield quantitative results. They therefore are not well suited for sensor-like applications meant to deal with all kinds of plant samples. However, by tailoring the method for a single well-defined application, TD-NMR can become simple and fast. An example of this is the Solid-Fat Content determination (SFC), currently the gold standard method to determine the ratio between liquid oil and solid fat in mixtures (Van Putte and Van Den Enden, 1974; van Boekel, 1981). A similar TD-NMR approach has been applied to estimate the solid to liquid fraction of tritium trapped in nano bubbles (Abell and Cowgill, 1991). A variant of the SFC method was devised to measure moisture or lipid contents in dry seeds (Guthausen and Kasten, 2005; Todt et al., 2006) and is used for seed phenotyping (Rolletschek et al., 2015; Melchinger et al., 2018), but is only valid for moisture contents of up to 15% (Todt et al., 2006), which is far below the range required for living plants.

In the current study we utilize a mobile, small scale NMR magnet that allows easy insertion of intact living plants or plant parts. It is fitted with an rf coil and tuning circuitry that possesses a dead time short enough to detect not only the NMR signal of protons in liquids, but also that of protons in solids (Mitchell et al., 2014). The setup is driven by a compact spectrometer, mounted in a temperature controlled housing to allow for use in environments with solar irradiation, moderate levels of moisture and variable temperatures. The housing is fitted with a lithium ion battery to allow for standalone operation. The NMR magnet and spectrometer together comprise an NMR relaxometer that is temperature insensitive and can easily be moved around.

To permit straightforward, sensor-like measurements and real-time data processing, we draw upon the principles of the SFC method, but modified it to allow operation with even the most basic of spectrometers and with mobile NMR magnets of limited field homogeneity. The method enables the detection of moisture contents in a range relevant for plants (15–100%) and correlates linearly with WC and DMC of plant organs as determined by gravimetric analysis. It thus provides a quantitative measure for the proton density of solids and liquids in plant samples and is coined Solid and Liquid matter Content determination (SLC). We validate the SLC method by demonstrating that it is inherently robust and allows accurate measurement of liquids (water) and solids (dry matter) in a variety of plant samples. A small scale NMR device, combined with the SLC method, thus makes for a sensor-like implementation of NMR on plants that is straightforward to use. We propose to call such devices NMR plant sensors. We demonstrate the utility of NMR sensing in an experimental context by monitoring changes in the water content of leaves of rice as affected by drought; and by monitoring the dynamics of water and dry matter accumulation in the wheat ear during the course of seed filling.

## Materials and Methods

### NMR Hard- and Software

#### Magnet and Magnet Housing

**FIGURE 1:**
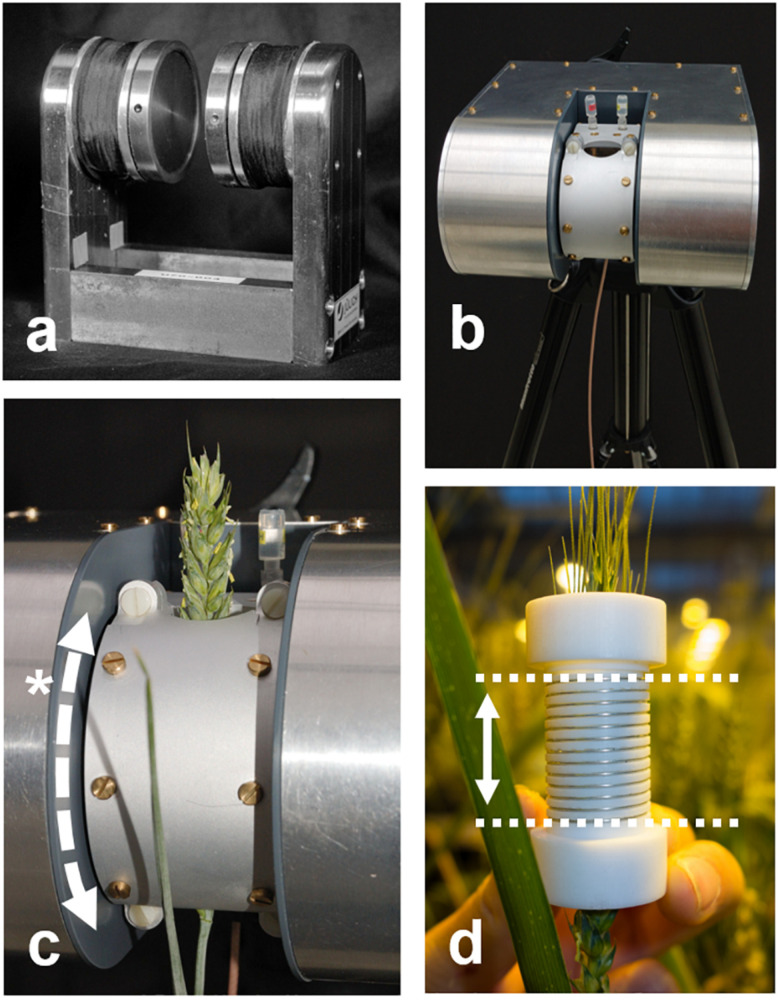
The NMR sensor presented here consists of a permanent C-shaped magnet **(a)**, offering unrestricted access to plants. The magnet is fitted with resistive heating wire around the poles and an accurate temperature controller. The magnet is placed in an insulating housing and mounted on a tripod **(b)** or adjustable stand. The probe housing **(c)** contains a solenoidal rf coil **(d)** and provides shielding against external rf signals. The probe housing can be opened and the rf coil can be split to allow access for long samples such as plant stems or branches. It further can be rotated around the magnet poles (*) to match the angle of the sample. The rf coil inside the probe housing allows for samples of up to 20 mm in diameter. The sensitive volume of the rf coil is as high as the coil is tall (d, dotted lines; maximum 25 mm). Convenient samples such as a wheat spike do not require the probe head to be opened, they easily slide in.

The basis of the NMR sensor was a 4.5 kg permanent C-shaped magnet, developed in-house (Figure 1a). The magnet yoke was constructed of soft steel, the poles of the magnets of grade 50 NdFeB permanent magnet material [remanence (Br) 1.42T; MCE, Bedfordshire, United Kingdom]. On the magnet poles (Ø 70 mm) soft steel pole caps were placed, consisting of a center piece with an axially adjustable outer ring. The magnet generated a field strength (B_0_) of 242 mT (10.3 MHz) over an air gap of 30 mm. At the edge of the insulating housing, closest to the probe holder, the remaining magnetic field was ∼ 50 mT; at a distance of 10 cm from the poles the magnetic field rapidly drops off to less than 2 mT. At this distance sensitive equipment such as the sensor head of a LiCor Li6400 gas exchange system can already be used without trouble. The homogeneity of the magnet was 88 ppm for a long sample completely filling a 15 mm coil, 34 ppm for a long sample measuring 5 mm in diameter.

NdFeB currently offers the highest field strengths and is affordable, but has a large temperature coefficient (Br −0.11% / °C). Small temperature changes thus cause large changes in resonance frequency. The sensitivity of the tuned coil can be assumed constant over at least ± 1 KHz from B_0_. To limit frequency drift to that range, magnet temperature should remain stable to ± 0.09°C. This requirement was met by placing the magnet in an aluminum housing (Figure 1b) filled with Styrodur insulation foam (BASF, Ludwigshafen, Germany). Resistive heater wire was used to heat the magnet, powered and regulated by a high precision temperature controller. Magnet temperature could be kept constant to within ± 0.1°C or better, if magnet set point temperature was at least 5°C higher than the highest diurnal temperature, but not more than 25°C higher than the lowest diurnal temperature.

#### Sample Holder and rf Coils

An aluminum probe head was constructed to accommodate the tuning capacitors, center the rf coil in the magnet and shield the coil against rf interference (Figure 1c). To allow branches or fruits into the magnet at any angle the probe head could rotate around the magnet poles (*). To enable mounting of the rf coils on stems or branches, Teflon coil formers were manufactured which could be split into two halves. The rf coils were wound by hand onto the formers, using 0.4 mm silver coated copper wire (Figure 1d). To facilitate winding a solenoidal groove was cut into the former. For the rice leaves a 6 mm (i.d.) coil with 15 turns was used, for all other samples a 15 mm coil and 13 turns. Coil height typically is chosen ∼1.3 times coil diameter for optimal performance, but can be chosen longer or shorter if required. The assembly of magnet, temperature controller and probe head together weighed 6.8 kg.

#### Spectrometer and Housing

A Kea II spectrometer with built-in 100 W rf amplifier (Magritek, Wellington, New Zealand) was mounted in a light weight 19 flight case (SKB, California, United States) together with a lithium ion battery (25.9 V, 12.6 Ah, Powerizer, United States), allowing 8 h of stand-alone use, depending on intensity of the sequences that are run. The output voltage of the battery was lowered to 24.0 V to match the requirements of the spectrometer. The case, spectrometer and battery together weighed 5 kg.

#### Sequences, Experimental Settings, and Data Processing

**FIGURE 2:**
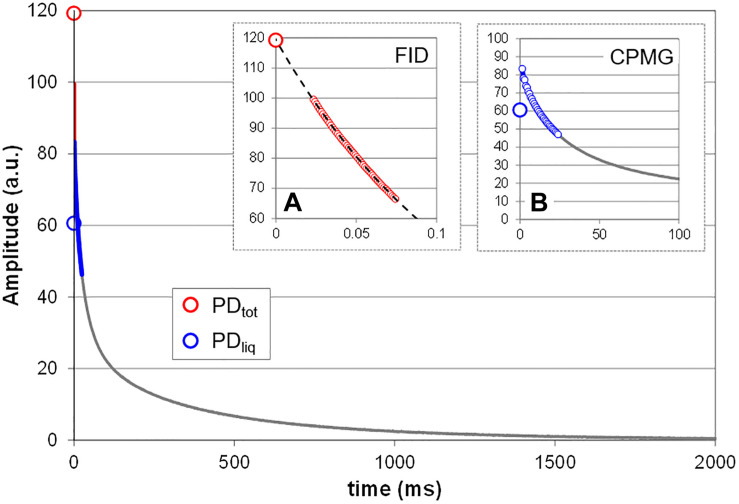
Measurement principle. Shown is the T_2_ relaxation curve of a mature bean pod. Two NMR methods (sequences) were combined to acquire the NMR signal, FID **(A)** and CPMG **(B)**. The FID sequence is used to acquire the signal of all protons in the sample, including that of the fast decaying protons in the solids, and provides an estimate of the total proton density (PD_tot_). To obtain this value, the FID data points between 0 and 75 μs (small red circles) are fitted with a single exponential and extrapolated to Y. To estimate the proton density of all liquids (PD_liq_) the data points of the CPMG curve between 0 and 25 ms (smaller blue circles) are averaged. The difference between PD_tot_ and PD_liq_ is a measure for the proton density of all solids in the sample (PD_sol_). Depending on the spectrometer and its software FID and CPMG can be acquired in a single measurement; in the current study the two were measured separately.

All measurements were done using the spectrometer’s proprietary software (Prospa, Magritek, New Zealand). Total proton density (PD_tot_, i.e., liquids plus solids; Figure 2) was measured by acquiring the free induction decay (FID) of the sample. PD_tot_ was estimated by mono-exponentially fitting the 0–75 μs interval of the FID (Figure 2A). Fitting signal amplitude against time, PD_tot_ is given by the Y intercept (120 a.u. in the shown sample). Note that this approximation only holds as long as it can be assumed that the sample does not contain a significant fraction of crystalline or glassy proton bearing solids, as could for example happen when seeds dry down to moisture contents below 15%. Such solids would add an even faster decaying fraction to the solid signal which, with the current dead time of the rf circuitry, would not or only partly be detected (van den Dries et al., 1998). In living plant tissue this condition normally is not met.

The proton density of the liquid fraction (PD_liq_; Figure 2) was approximated on the basis of a Carr-Purcell-Meiboom-Gill (CPMG) type measurement (Edzes et al., 1998). The average CPMG amplitude over the interval 0–25 ms was calculated to approximate PD_liq_ (Figure 2B). This interval was chosen because we empirically found it to provide a linear relationship with water content for the widest variety of plant tissues, ranging from herbaceous leaves to living wood. The proton density of the solid fraction (PD_sol_) is equal to the difference between PD_tot_ and PD_liq_.

The NMR settings that were used for the experiments and for the acquisition of the reference curves are summarized in Table 1.

**TABLE 1:** NMR settings.

	Rice leaves		Wheat ear, reference samples			
Sequence	CPMG		FID		CPMG	
Repetition time	6 s		5 s		5 s	
Nr. of averages	32		32		32	
Echo time	500 μs		n.a.		750 μs	
Nr. of echoes	6,000		n.a.		1,500	
Spectral width	100 kHz		1 MHz		100 kHz	
Points acquired	8 pts/echo		512		8 pts/echo	
90° pulse	8 μs	−11.5 dB	4 μs	−4.25 dB	16 μs	−10.75 dB
180° pulse	8 μs	−17.5 dB	n.a.	n.a.	16 μs	−16.75 dB

### Plant Material and Handling

#### Rice

Rice plants (*Oryza sativa* cv. Nuovo Maratelli) were sown in potting soil (ED 73, Stangenberg GmbH, Germany) and grown in a greenhouse (25°C day, 18°C night, RH 65%). Material for reference curves was collected 2 months after sowing. Batches consisting of the youngest and oldest green and fully developed leaves were harvested and cut into 4 cm pieces. Sets of two pieces were gently folded into a stack of 4 lamina to make a single sample, taking care not to crush the leaves by folding too sharply. The samples were subsequently loaded into a thin walled glass test tube, weighed, immediately measured by NMR, left to dry at room temperature for 10 min under a slow continuous flow of air, fed into the tube by a Pasteur pipette, and weighed and measured by NMR again. This cycle was repeated several times for all samples. At the end of the experiment the leaves were dried at 85°C for 24 h and weighed to calculate DW.

For an osmotic stress experiment rice seedlings were placed in continuously aerated hydroculture in a climate chamber (day/night: 16 h/8 h; 24°/24°C, RH 65%, 200 μmol m^2^/s). The shoots were secured in a polystyrene carrier with soft polyurethane foam. The plants were grown in ½ strength modified Hoagland solution (6.5 mM KNO_3_, 4 mM Ca(NO_3_)_2_, 2 mM NH_4_H_2_PO_4_, 2 mM MgSO_4_, 4.6 μM H_3_BO_3_, 0.5 μM MnCl_2_, 0.2 μM ZnSO_4_, 0.1 μM (NH_4_)_6_Mo_7_O_24_, 0.2 μM CuSO_4_, 45 μM Fe-EDTA). The nutrient solution was refreshed every 3 days. The plants were kept vegetative by removing panicles as soon as they emerged. Rapid step-wise osmotic challenges were induced by adding 50, 100, 150, or 200 g/L polyethylene glycol (PEG 6000, BioChemica, AppliChem GmbH, Germany) to the nutrient solution, giving rise to osmotic potentials of −0.07, −0.18, −0.33, and −0.54 MPa, respectively (Michel and Kaufmann, 1973). Prolonged exposure to a concentration of 200 g/L was lethal.

Of each plant, three leaves were bundled and threaded through the coil of the NMR plant sensor. The largest part of the leaves protruded out of the sensors and was exposed to the light, whereas at the position of the NMR sensor the leaves were fully shaded (see Figures 1c,d). Of each plant, transpiration of one of the leaves was measured by means of a portable photosynthesis system (LI6400 XT, Li-Cor, Lincoln, NE, United States). Inside the cuvette, the leaf was illuminated by means of the built-in LEDs and exposed to environmental conditions mirroring those in the climate chamber (light, CO2, and humidity).

#### Wheat

Summer wheat (*Triticum aestivum* cv. Anza) was hydroponically grown in a growth chamber (day/night: 14 h/10 h, 21/17°C, 85/70% RH). The plants were sown in vermiculite and 1/5 strength Hoagland solution, after 1 week transferred to hydroculture in ½ strength, continuously aerated modified Hoagland solution (5 mM MES-KOH, pH 5.3, 2.5 mM Ca[NO_3_]_2_, 2.5 mM KNO_3_, 1 mM MgSO_4_, 0.5 mM KH_2_PO_4_, supplemented with micronutrients at a final concentration of 0.09 mM Fe-EDTA, 50 μM H_3_BO_3_, 10 μM MnCl_2_, 1 μM ZnSO_4_, 1 μM CuSO_4_, 0.5 μM Na_2_MoO_4_). Tillers were removed as soon as they appeared, leaving only the main stem and its spike.

For the experiment vigorously growing plants at booting stage were selected. The NMR plant sensor was set up in a small climate chamber (Sanyo MLR-350H; 16 h day/8 h night; 23°/23°C, RH 70%; 250 μmol m^–2^ s^–1^ PAR) on a custom built aluminum support, high enough to slip the wheat ear into the sample holder without bending the main stem or rachis. The pot of the plant was placed on a lab jack to allow fine adjustments of height. To prevent the ear from shifting upwards through the coil a small Ø 0.20 mm copper rod was threaded through the bottom of the ear, between the pedicels of the spikelets, perpendicular to the axis, and directly underneath the rf coil. As the stem elongated, the ear thus fixed itself relative to the coil. The lab jack was lowered during the growth period whenever needed to prevent buckling of the stem,.

To collect material for a reference curve, plants were sown each week over a period of 6 weeks. Ears of all stages of development were harvested as soon as the oldest ears started to ripen. Sections of 15 mm were excised from the middle of the ears. The pieces were weighed, measured in the NMR plant sensor, and dried overnight in an oven at 90°C to obtain their dry weight. To mimic what the sensor would measure in the intact ear the whole section was measured, including chaff and rachis material.

#### Plant Materials for Reference Curves: Bean, Pea, Oak

Bean (*Phaseolus vulgaris*) and pea (*Pisum sativum* cv. Cayanne): Sets of 3 seeds were sown in 5 l pots with potting soil (ED 73, Stangenberg GmbH, Germany), 5 pots in total. After germination the least vigorous seedlings were culled, leaving 1 plant per pot. The plants were grown in a greenhouse (25°C day, 18°C night, RH 65%, light intensity 300–1,200 (μmol m^–2^ s^–1^). Pods for reference curves were collected randomly, between 2 and 3 months after sowing, as soon as the plants had pods in all stages of development [2 days after anthesis (DAA)–mature]. Sections of 15 mm were excised from the middle of the pods. The pieces were weighed, measured in the NMR plant sensor, and dried for 48 h in an oven at 90°C to obtain their dry weight.

Oak (*Quercus robur*) samples were excised from the main stem of 4 year old saplings purchased at a local nursery. They were grown outdoors for 2 months and kept well-watered by means of an automated watering system. A 10 cm long sample was harvested in early summer and put in the NMR plant sensor, with the sample resting on an automatic balance in a non-magnetic support, without touching the coil assembly. During dry down its weight was recorded continuously.

### Pressure-Volume Analysis

Fully expanded leaves were collected from well-watered rice plants (*Oryza sativa* cv. Nuovo Maratelli) in the dark. The leaves were put in water until saturated and weighed. Of three leaves the water potential was determined without prior drying; subsequent leaves were progressively benchtop dried and weighed to determine the amount of water lost. Water potentials were determined by means of a pressure bomb (Model 1000, PMS Instrument, United States), according to the method by Scholander et al. (1964). Before measurement the leaves were enclosed in plastic bags; pressure volume analysis was done as described by Koide et al. (1989).

## Results

### Validation SLC Determination: Independence of Coil Load and Sample Position

Basic requirements for the stability of the SLC method are (I) that PD_liq_ and PD_tot_ scale linearly with the number of protons in the coil, (II) the signal response is linear and not affected by changes in coil load, and (III) the signal amplitude remains constant regardless of the position of the sample in the coil.

To test the linearity of the response of PD_tot_ and PD_liq_ as a function of the amount of material in the coil (i.e., loading ratio) a test tube was incrementally filled with a dilute CuSO_4_ solution with a T_2_ of 200 ms. Both PD_tot_ and PD_liq_ were found to scale linearly with sample volume (Supplementary Figure S1). The slope of both traces, as well as the ratio between the two values remained constant, demonstrating that PD_tot_ and PD_liq_ are not affected by changes in coil load. Due to the way the two values are determined (Figure 2), PD_tot_ will always be higher than PD_liq_, even if all protons are in liquids.

Independence of radial sample position was tested by loading long capillaries filled with reference liquid into the coil. To simulate long slender plant organs such as cereal ears or leaves, the filled part of the capillaries extended well outside the coil. They were placed in random positions inside the coil until the coil was filled to capacity (34 capillaries). The results match those of Supplementary Figure S1, demonstrating that coil load and sample position do not affect the linearity of the response of the NMR sensor (Supplementary Figure S2).

### Validation SLC Determination: Reference Curves

To test the linearity of the response of PD_tot_, PD_liq_, and PD_sol_ in samples of varying origin and solid matter content, reference measurements were acquired for model samples (cooked rice and starch suspended in agarose), plant samples in progressive stages of dehydration (oak stem and rice leaves) and reproductive organs in various stages of development (pea pod, bean pod, and wheat ear).

**FIGURE 3:**
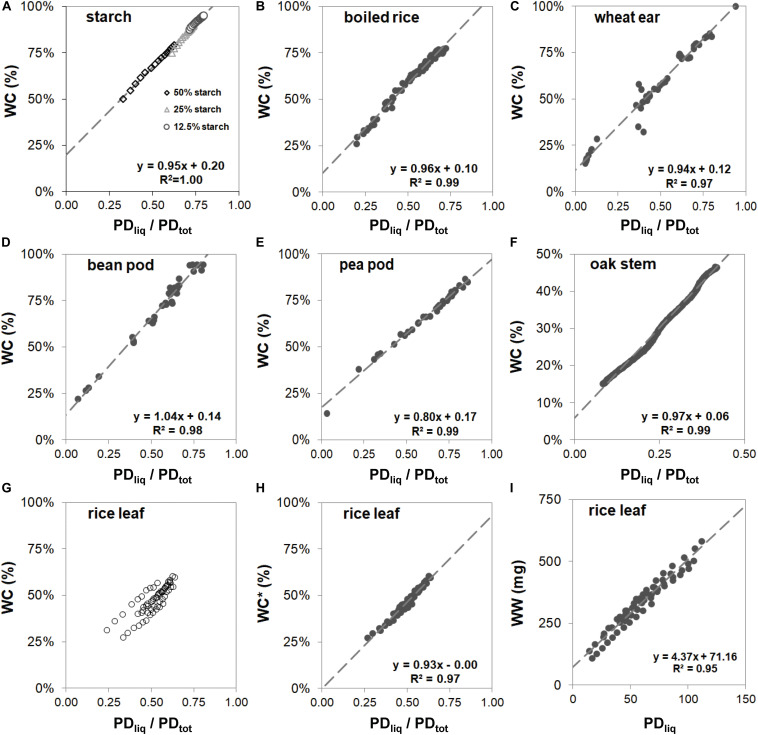
Reference curves for maize starch in agarose gel **(A)**, boiled rice **(B)**, wheat spikes harvested from booting stage onwards throughout development **(C)**, bean pods harvested at progressive stages of development **(D)**, pea pods harvested at progressive stages of development **(D)**, stem of oak **(E)**, and rice leaves **(G–I)**. In figures **(A–G)** water content (WC) is plotted against PD_liq_/PD_tot_. In panel **(G)** 9 strings of points are plotted, each string representing samples of a separate rice leaf. The three oldest leaves show up as strings with deviating offsets. The data points from each leaf sample were fitted individually and the offset value **(C)** subtracted from the data points to plot normalized WC* against PD_liq_/PD_tot_ **(H)**. In plot **(I)** water weight (WW) of rice leaf samples is plotted against PD_liq_.

Despite the differences in cellular- and micro-structure of the samples, and the variation in the methods that were used to obtain samples of varying dry matter content (for the model samples by addition of water; for the woody samples and leaves by gradual desk top dry-down; for fruits and seeds by harvesting at various stages of fruit development), the reference curves of all samples exhibited a tight linear relationship between WC (i.e., water weight relative to total sample fresh weight) and PD_liq_/PD_tot_ (Figure 3), except that of rice leaf (Figure 3G). The slopes of the linear fits in graphs 3a through 3f varied between 0.80 (pea pod, Figure 3E) and 1.04 (bean pod, Figure 3D). For these samples, the parameters of the linear fits can be used to calculate WC in subsequent measurements:

(2) $$WC=a\cdot \left(\frac{{\text{PD}}_{\text{liq}}}{{\text{PD}}_{\text{tot}}}\right)+c,$$

where *a* is the slope and *c* is the offset of the linear regression.

The correlation between WC and PD_liq_/PD_tot_ for rice leaf (Figure 3G) at first glance seems less tight than that of the other samples. Here the plot consists of nine strings of points, each with the same slope, but a slightly different offset. Each string of points originated from a single drying sample; every sample contained leaf material of a single detached leaf. The leaves were chosen from random positions on the plants, comprising three young mature and three old leaves. The older leaves became visible as outliers, one with a higher offset than the young mature leaves, and two with a lower. Differences in offset not only became visible when plotting WC against PD_liq_/PD_tot_, but also when plotting it against PD_liq_ (Figure 3I). Here, too, older leaves gave rise to a slight offset.

Despite the differences in offset (Figure 3G), the slope of the response of the drying rice leaf samples remained virtually identical. To visualize this, the data points from each leaf piece set (sample) were fitted individually and the offset value (c) detracted from the data points to obtain a normalized WC^∗^. Plotted against that, PD_liq_/PD_tot_ was found to again correlate tightly and linearly with WC^∗^ (Figure 3H). In the other plant samples in Figure 3, differences in age or developmental stage did not result in variations in offset.

### Water Content of Rice Leaves Exposed to an Osmotic Challenge

**FIGURE 4:**
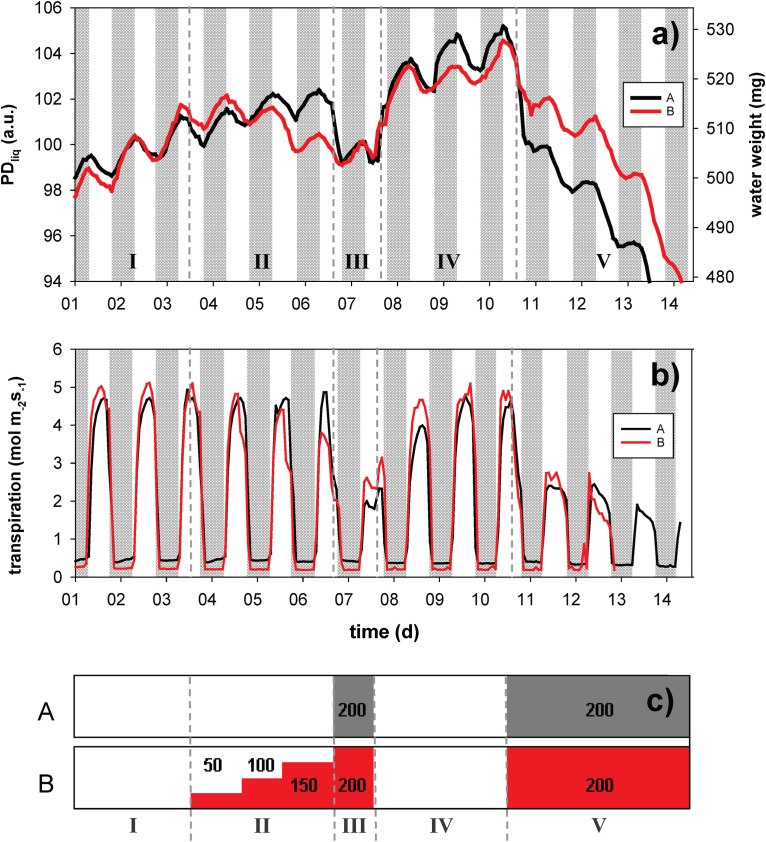
Dynamic changes in leaf water content, expressed as liquid proton density (PD_liq_, left axis) and water weight (WW, right axis), of sets of three leaves of rice plants grown in hydro culture **(a)**. The plants were exposed to PEG 6000 induced changes in root medium osmotic potential, either in a shock-wise (black trace, A) or step-wise fashion (red trace, B). PD_liq_ is expressed in arbitrary units (a.u.). The experiment lasted for a total of 14 days; night periods are indicated in gray. Leaf transpiration (mol m^2^ s^– 1^) was continuously monitored on the same plant by means of a LiCOR 6400 **(b)**. The timing of the osmotic treatments is visualized in window **(c)**; the PEG treatment is given in g/L. The highest concentration (200 g/L) equates to an osmotic potential of –0.54 MPa, which for rice is considered strong osmotic stress.

To explore the utility of the NMR plant sensor to monitor the WC of growing leaves as affected by drought, salinity or transpiration, we challenged hydroponically grown *Oryza sativa* (cv. Nuovo Maratelli) with shock-wise (group A) and gradual (group B) changes in the osmotic potential of the nutrient solution (treatment sequence and severity indicated in Figure 4c). During the 14 day experiment NMR sensors were mounted at the base of a set of 3 leaves, shading the lower 10 cm. To monitor starch buildup and remobilization in this deep shaded part of the leaves was not considered useful. We thus chose to only measure PD_liq_, not PD_sol_. Not measuring PD_tot_ (FID sequence), allowed higher time resolved measurements of PD_liq_ (CPMG sequence).

In phase I (before osmotic challenge) the leaves of both groups showed continuous expansion growth in width and/or thickness (please note that leaf expansion in length would not be detected), with superimposed a diurnal pattern of water loss (in the light) and uptake (at night) (Figure 4a). In both treatments (A and B) between 0.8 and 0.9% of leaf water was lost during the day, whereas between 1.4% (A) and 2.0% (B) was gained in the dark (Figure 4a). The pattern of daytime water loss and refilling inversely matched the pattern of leaf transpiration (Figure 4b).

In phase II and III osmotic challenges were imposed (Figure 4c). Under osmotic stress the diurnal variations became smaller, but remained clearly visible. Both the gradual and the shock-wise challenges were instantaneously reflected in leaf water content. In phase III, where both groups were challenged with 200 g/L PEG, PD_liq_ of both groups was approximately identical. Group B, however, was able to maintain a higher transpiration rate (Figure 4b), potentially indicating a degree of osmotic adjustment. Upon transition from phase II to III (exposure from 0 to 200 g/L PEG), group A rapidly lost water (∼2.3%), whereas in group B the switch from 150 to 200 g/L PEG did not even become visible as a clear breakpoint in the trace any more. In phase IV the osmotic challenge was lifted, allowing the plants to recover for 3 days. In this phase both treatment groups rapidly regained water and resumed growth. In the final phase (V) both treatment groups were again exposed to 200 g/L PEG for 4 days. Both groups experienced a rapid decline in leaf water, but group A in the first hours (until light off) lost water more than twice as fast as group B (∼3.3 vs. 1.4%).

### Rice Leaf Water Content vs. Leaf Water Potential

**FIGURE 5:**
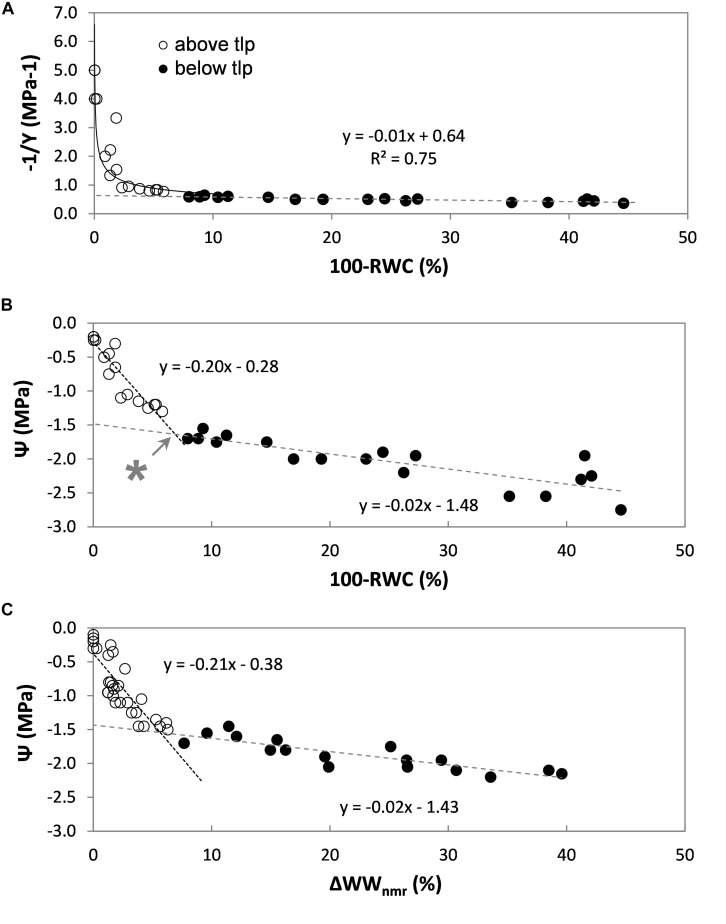
Pressure-volume analysis of a set of rice leaves (*Oryza sativa*, cv Nuovo Maratelli). Conventionally the inverse of the leaf water potential (1/Ψ) is plotted against the change in leaf relative water content (100-RWC) **(A)**. Open symbols (∘) represent data points above tlp, closed (∙) below (all panels). To also directly visualize the correlation between Ψ and leaf water content, Ψ is plotted against loss in RWC in plot **(B)**. The turgor loss point (tlp) was reached at –1.6 MPa, which corresponded with a decrease in leaf RWC of 7% (*). In plot **(C)** the same relationship is plotted, but acquired from a second set of leaves, excised from a second set of plants. Here the relative loss of water was measured by means of NMR (ΔWW_*nmr*_). In all plots, every data point represents a different leaf.

In order to relate changes in leaf WC to changes in leaf water potential (Ψ_leaf_) as measured by means of the Scholander pressure chamber (Scholander et al., 1964), NMR plant sensor readings can be combined with pressure-volume (p-v) curve analysis (Figure 5). In the p-v method conventionally the inverse of the leaf water potential (1/Ψ) is plotted against loss of leaf relative water content (100-RWC) (Figure 5A). RWC in this context is defined as the ratio of the water weight at time t (WW), relative to the water mass when saturated with water (WW_sat_):

(3) $$RWC=\frac{WW}{WW_{\text{sat}}}$$

The turgor loss point (tlp) is defined as the point at which the curve becomes linear [for details, see Koide et al. (1989), Bartlett et al. (2012b)]. For rice Ψ_*tlp*_ was reached at −1.6 MPa (Figure 5A).

Above tlp, leaf water potential (Ψ_leaf_) is determined by the (turgor) pressure potential (Ψ_*p*_) and the osmotic potential of the leaf symplast (Ψ_*s*_): Ψ_leaf_ = Ψ_*s*_ + Ψ_*p*_. The most dominant factor is Ψ_*p*_, which can be expected to decrease linearly with RWC (Bartlett et al., 2012a). Below tlp, turgor pressure usually is assumed to be 0 and can be neglected; Ψ_leaf_ then would be solely determined by Ψ_*s*_. For our purposes the bi-phasic correlation between Ψ_leaf_ and 100-RWC above and below tlp was satisfactorily characterized by two linear regressions (y = *a*x + *c*, Figure 5B). The intersection between the two regression lines indicates tlp, at which point the decrease in leaf RWC was approx. 7% (Figure 5B, indicated with ^∗^).

### Seed Filling Characteristics of a Developing Wheat Ear

**FIGURE 6:**
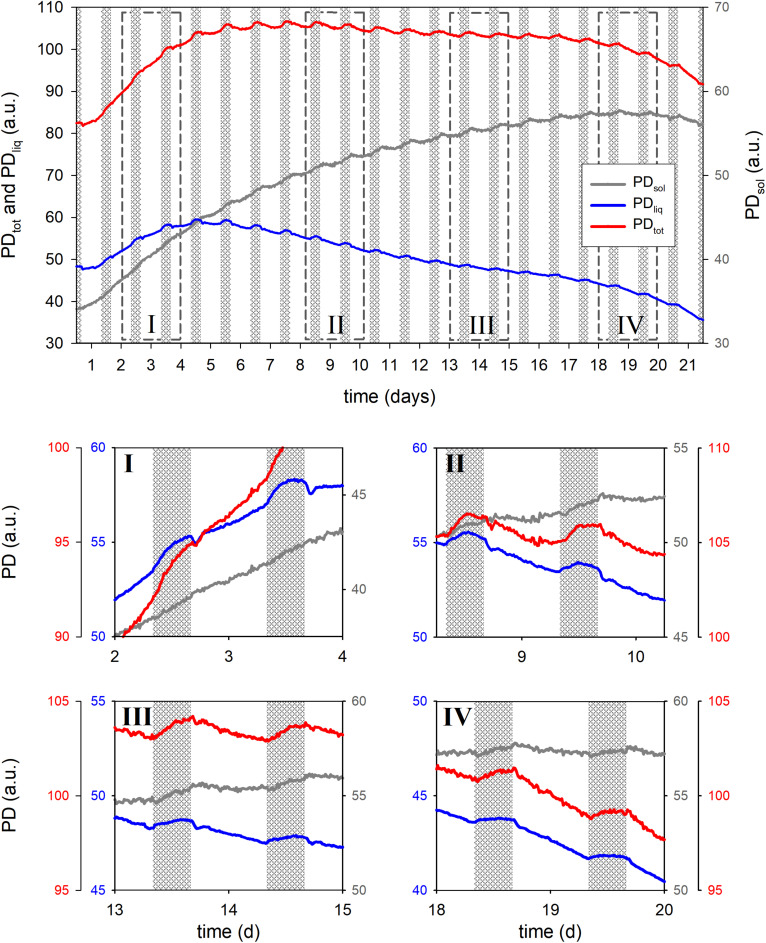
Total proton density (PD_tot_), liquid proton density (PD_liq_) and solid proton density (PD_sol_) of a developing wheat ear during 21 days of growth. Detailed views of the growth curve are given in boxes I through IV, dashed boxes in the main graph indicate the positions. In the main graph the *Y*-axes for PD_tot_ and PD_liq_ are displayed at the **left** side of the graph, the *Y*-axis of PD_sol_ at the **right**. In the detail graphs every trace has its own *Y*-axis, the colors of the axes match the colors of the traces.

For a second example we monitored the seed filling dynamics of a wheat ear from booting until physiological maturity (Figure 6). PD_tot_ was found to top out on day 7 and PD_liq_ even earlier, reaching a maximum in the night of day 4. After that PD_liq_ in the ear slowly decreased while PD_sol_ steadily increased, reaching its maximum on night 18.

In the last days of the experiment a slight decline in PD_sol_ was observed. This may indicate a relocation of solids from the tissues surrounding the seeds (glumes, rachis etc.) to the seeds and vegetative parts of the plant. Methodical limitations may, however, also have played a role. As seeds mature and dry, starch will at some point turn crystalline. This phase transition would further decrease the already extremely short T_2_ relaxation time of such solids. Currently the dead time of the rf circuitry of the NMR sensor prohibits an adequate sampling of such short T_2_ signals, which may lead to an underestimation of PD_sol_ as seeds dry out. In future versions of the sensor this limitation will be alleviated.

PD_liq_ fluctuated markedly in response to light on–light off events, losing up to 2% (comparing the last night value to the lowest early morning value) when the light was turned on, but compensating that loss upon light off. Most uptake of water took place in the first 5 h of the night, especially during the first 10 nights. In the last 5 h of the night PD_liq_ either remained constant, or declined (all developmental stages except night 2).

In the earlier stages of development light-on events gave rise to a dampened oscillatory response in PD_liq_ (Figure 6, box I through III). In the first stages of seed development only a single oscillation was observed (week 1, Figure 6, box I), but in week 2 (Figure 6, box II) the oscillations persisted throughout the day. In week 3 the oscillations appeared to lessen and no longer lasted the entire day (Figure 6, box III), in week 4 the oscillations finally disappeared (Figure 6, box IV: day 20).

**FIGURE 7:**
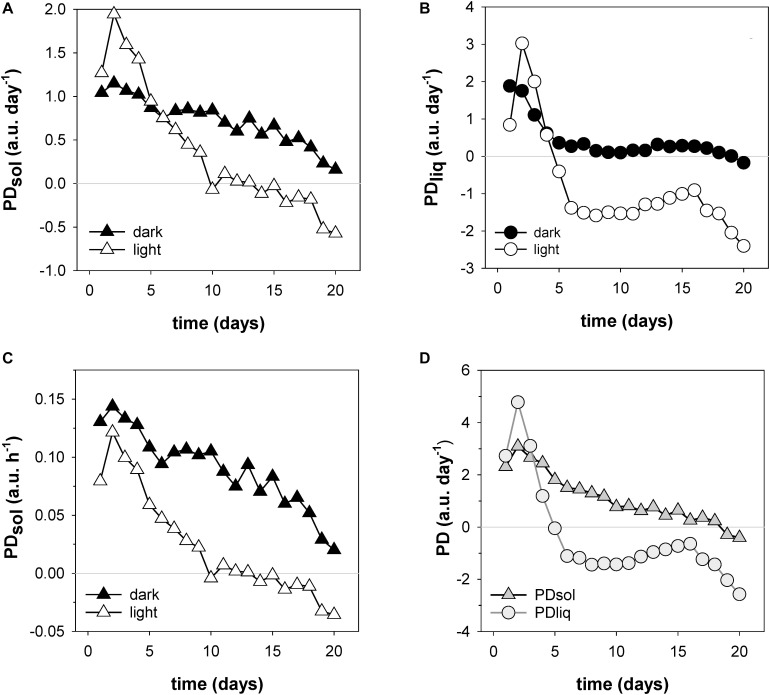
Filling rates of solids (PD_sol_) and liquids (PD_liq_) in a developing wheat ear. Shown are day and night time accumulation rates of PD_sol_ **(A)** and PD_liq_ **(B)**, expressed in a.u. per day (24 h period); day and night time solid accumulation rates expressed in a.u. per hour **(C)**; and total dry matter and liquid accumulation rates per 24 h day **(D)**.

A unique and especially useful feature of the NMR sensing approach is that it enables the non-invasive determination of dry matter accumulation rates (Figure 7). In the first week dry matter accumulation occurred day and night (Figure 6, box I; Figures 7A,C), but from day 10 onwards PD_sol_ remained virtually stagnant during the day, increasing only at night (Figure 6, box II–IV; Figures 7A,C). From day 6 the ear started to lose water during the day; after day 16 the ear started to lose water even more quickly (Figure 7B). From day 19 onwards, as the spike dried out, PD_sol_ started to decline as well (Figure 7D). Since the light period was twice as long as the dark (16 vs. 8 h), the differences between day and night time dry matter deposition are larger than they appear on the basis of their relative accumulation rates alone (c.f. Figures 7A,C). Cumulatively, more than two thirds (68%) of the dry matter in the ear was deposited during the night, only one third (32%) during the day (Figure 7C).

## Discussion

### NMR Plant Sensor: Hardware

A basic, but temperature stable small-scale NMR device combined with the SLC determination suffices to construct an NMR plant sensor with which the total, liquid and solid proton densities of living plants can be measured. The NMR magnet that makes up the sensor head (Figure 1) can be robust and affordable and can, in principle, be scaled up or down to meet the size requirements of any sample. The weight of the magnet will, however, scale with roughly the 3rd power of its size.

The NMR sensor used in this study was compact in size and could already be moved with ease, but potential for further downsizing remains. Firstly, for the spectrometer. The SLC approach not only reduces the complexity of data evaluation, but also reduces the number of data points acquired per excitation. Tailored to these relaxed requirements the spectrometer can become smaller, simpler and cheaper. Designs for small, low-cost and often open-source spectrometers are readily available (e.g., Takeda, 2011; Chen et al., 2015; Michal, 2018; Ariando et al., 2019). Such spectrometers could become the core of an NMR plant sensor if fitted with an rf amplifier of sufficient power. Secondly, other magnet concepts may be employed to construct the sensor head. In our study we used C-shaped magnets, as they allow open access from the side and can easily be re-adjusted for optimal homogeneity. The latter might be necessary after temperature cycles, shocks or bumps during transport or use. However, more complex homogeneous magnet designs such as openable or fixed Halbach magnets (Windt et al., 2011; Blümler and Casanova, 2016) may also deserve consideration, especially if large magnets with low weights are required. Unilateral magnets with inhomogeneous fields (Capitani et al., 2009; Danieli and Blümich, 2013) would not be suitable for the task as they typically do not allow the acquisition of FID’s of sufficient quality to determine PD_tot_.

### NMR Plant Sensor: SLC Determination

The basis of the SLC approach is the empirical correlation between PD_tot_, PD_liq_, and PD_sol_ as measured by NMR, and water and solid matter content as measured by weighing and oven drying. For all samples this correlation was linear and robust (Figures 3A–F), exhibiting only a slight variation in slope and offset between different materials, organs and species. Rice leaf was an exception, in that samples from older leaves dried down with different offsets than the younger ones (Figure 3G). The slopes of the individual rice leaf samples, however, were virtually identical (Figure 3H).

The variation in angles and offsets illustrates the necessity to measure a reference line for every type of sample. In theory, four physicochemical sample properties could affect the angle and offset of the reference line in the SLC determination. First, dissolved organic molecules (e.g., soluble sugars, small proteins) will add to PD_liq_, but after oven drying will be weighed as solid matter. Second, inorganic matter in the sample will add to DW, but will not be detected by NMR. Third, water bound in tight matrices such as dense cell walls, or in and between starch kernels, may exhibit very short T_2_ relaxation times and thus add to PD_sol_. Differences in sample microstructure may affect how much water is locked in such matrices. A fourth property is variation in the organic solid matter composition of samples: the molecular proton density (number of protons per gram organic matter) may vary between organic molecules.

Given the number of properties that theoretically could influence them, the correlations between WC and PD_liq_/PD_tot_ are exceptionally linear and tight (Figure 3). If the offset of the reference line can be assumed to remain constant between samples, then a single SLC measurement would be sufficient to determine FW, WW, WC, or DMC of a sample. However, as is exemplified by the rice leaf example, some samples may exhibit more spread and variation than others. It is important to be aware of such deviations when interpreting the results of the SLC determination. The best way to ensure the validity of the reference curves will therefore be to generate them on the basis of samples that have experienced the same, or a comparable treatment as the samples in the actual experiment.

### Monitoring Leaf Water Content and Plant Water Status: Rice Osmotic Challenge

In the rice osmotic challenge experiment the usability of the NMR plant sensor to monitor leaf and plant water status is demonstrated. PD_liq_ linearly correlates with changes in WW (Figure 3I):

(4) $$\text{WW}=a\cdot {\text{PD}}_{\text{liq}}+c.$$

Here *a* and *c* represent the slope and offset of the fitted regression line, respectively. Combined with p-v data of the same organ, changes in WW (ΔWW) as measured on the basis of PD_liq_ may be used to approximate leaf- and stem xylem water potential of the intact plant. ΔWW can be calculated:

(5) $$\Delta \mathrm{WW}=\frac{{\text{PD}}_{\text{liq},\text{t}}+c}{{\text{PD}}_{\text{liq},{\text{t}}_{0}}+c}\cdot 100\%,$$

where PD_liq.t_ and PD_liq.t0_ represent PD_liq_ at time t and time t = 0, respectively. RWC, one of the cornerstone parameters to describe plant water status (Martinez-Vilalta et al., 2019), may also easily be obtained using the same relationship, if a value for proton density at full turgor (PD_liq.sat_) can be obtained (see Eq. 3):

(6) $$\text{RWC}=\frac{{\text{PD}}_{\text{liq},\text{t}}+c}{{\text{PD}}_{\text{liq},\text{sat}}+c}\cdot 100\%,$$

On the basis of ΔWW the change in leaf water potential (ΔΨ_leaf_) can then be approximated (Figure 5):

$$\Delta \Psi_{\text{leaf}}\left(MPa\right)=-0.20\cdot \Delta WW-0.28\mathrm{above}\mathrm{tlp},\mathrm{and}$$

$$\Delta \Psi_{\text{leaf}}\left(MPa\right)=-0.02\cdot \Delta WW-1.48\mathrm{below}\mathrm{tlp},$$

where the slope and offset values were taken from the linear regressions of the p-v curve in Figure 5.

At predawn, when in the absence of transpiration the plant is assumed to be at equilibrium with the soil, Ψ_leaf_ will be equal to the Ψ of the root medium (Ψ_soil_). It is reasonable to assume that this assumption holds for rice, even though for large or drought resistant woody species, it may not always do so (Améglio et al., 1999). When the soil is well-watered, Ψ_soil_ can be assumed to be 0. Predawn Ψ_leaf_ thus will approach 0 as well, and the relation above can be used to approximate Ψ_leaf_ in absolute terms: ΔΨ_leaf_ = Ψ_leaf_.

Having established the correlation between PD_liq_, WW and Ψ_leaf_, it becomes possible to quantify the rice leaf responses to the osmotic challenges. In treatment A, the shock-wise application of PEG decreased the water potential of the root medium to −0.54 MPa. Upon the transition from phase II–III WW decreased from 517.2 to 507.2 mg, a decrease of 1.7%. This is still far removed from tlp (ΔWW −7%). If it is assumed that in phase II, for treatment A, Ψ_leaf, predawn_ approximated 0, then in phase III Ψ_leaf, predawn_ may be estimated to have been −0.62 MPa, a value close to Ψ_medium_. (In group B the change in Ψ_leaf_ during the transition from phase II–III could not be estimated as the plant was already exposed to PEG in phase II). Interestingly, during the osmotic challenge of phase III transpiration of treatment B was markedly higher than that of treatment A, and upon release of the osmotic challenge in phase IV it recovered much quicker.

The second time that both plant groups were exposed to a −0.54 MPa root medium, in the transition phase IV–V, treatment A responded more strongly to the challenge than did treatment B. In treatment B ΔWW was −1.94%, equating a Ψ_leaf, predawn_ of −0.67 MPa at the end of the first night of phase V, and still close to Ψ_medium_. In treatment A the leaves lost 3.78% WW, equating a Ψ_leaf_ of −1.04 MPa. This is much lower than Ψ_medium_, suggesting that the plant could no longer cope with the challenge and failed to sufficiently replenish water at night to make up for the amount of water lost during the day – even though at that moment the transpiration rate of treatment A was lower than that of treatment B. In the subsequent days the plants kept losing water rapidly, only partially refilling during the night. In treatment B, ΔΨ_leaf_ did not decrease as quickly as did the plants in treatment A, while at the same time maintaining a higher transpiration rate (day 11). On the following day treatment B still exhibited a higher WW, but now with a transpiration rate lower than that of treatment B. Relative to the predawn value of day 10 (phase IV, osmotic challenge lifted), treatment A exceeded 7% loss of WW on day 12, passing tlp. Treatment B, in comparison, exceeded tlp 1 day later. In keeping with prior studies (Cutler et al., 1980; Steponkus et al., 1982), this confirms the hypothesis that a gradual exposure to an increasing osmotic challenge improves the plants ability to react to osmotic challenges; an ability that may be linked to osmotic adjustment (Steponkus et al., 1982; Pandey and Shukla, 2015), but also would appear to be linked to the acquisition of improved control over stomatal conductance.

### Monitoring Dry Matter Accumulation: Wheat Seed Filling Experiment

The wheat experiment demonstrates how NMR sensing can be used to monitor seed filling and dry matter accumulation in the living plant (Figures 6, 7). Many studies presented data on the development of FW, DW and WC during seed filling in wheat (Brenchley and Hall, 1909; Schnyder and Baum, 1992; Altenbach et al., 2003; Yang et al., 2004; Pepler et al., 2006; Xie et al., 2015; Neghliz et al., 2016; Zhang et al., 2017) or on seed filling in general (Kermode and Finch-Savage, 2002), but all used destructive methods. The pattern of development in FW, DW, and WC that we measured (Figure 6) matches well with these studies, but were obtained non-destructively and with a much higher time resolution: 4 min per data point, continuously, vs. only a single data point per day, in the daytime, for most other studies. To acquire even a limited number of data points per day with destructive techniques would require outrageously large numbers of plants. Using an NMR plant sensor, a limited number of plants already suffice for a comparison of genotypes and treatments, and require much less manual labor in harvesting, weighing and drying. We envisage that this will facilitate the comparison of seed developmental traits such as seed filling duration, maximum filling rate or average filling rate.

Apart from demonstrating the feasibility of the method, the experiment yielded two striking observations. The first is that most dry matter was deposited during the night, not the day. During the first week of seed filling dry matter deposition still occurred day and night, but in the second week the daytime contribution dropped strongly and became lower than that at night (Figures 6, 7). From day 10 onwards the daytime contribution became negligible. Cumulatively, throughout development approximately two thirds of the dry matter deposition took place during the night, even though the night periods lasted only half as long as the day (8 vs. 16 h). This finding is unexpected. Export of photosynthates from leaves most likely will have continued day and night (Jenner and Rathjen, 1972; Gordon et al., 1980; Graf and Smith, 2011), but even if the rate would have remained constant throughout the diurnal cycle, the short night time period would have caused the majority of carbohydrates to have been exported during the day. How, then, can it be explained that most solids in the ear accumulated during the night?

Perhaps the most likely explanation is that photosynthates exported from leaves were not, or only in part, routed directly toward the ear. Along the transport pathway to the ear, in leaf sheath, stem and pedicel, sizeable transient carbon storage pools are present (Kühbauch and Thome, 1989; Wardlaw and Willenbrink, 2000; Tadashi et al., 2001). It has been proposed that these reserves do not only provide carbon during the last stages of seed filling, but also maintain carbon supply to the grain when translocation of current photosynthates is insufficient to meet the needs of grains and of other sinks. This could comprise a low-capacity fast response, as well as high capacity long-term storage system (Wardlaw, 1990; Schnyder, 1993). Numerous studies provided evidence that storage and remobilization of carbon from short and long term storage pools indeed have a pronounced influence on the source-sink distribution and re-distribution of photosynthates in wheat (Schnyder, 1993 and references therein). Austin et al. (1977) showed that roughly half the photosynthate that ultimately reached the ear, took more than 10 days to get there. This suggests that the diurnal pattern of dry matter accumulation in the ear reflects not only export of newly fixed photosynthates from the leaves, but also fluctuating relative changes in sink strength between the ear and the various storage pools. This interpretation agrees well with the observation that the activity of enzymes associated with starch synthesis in the grains of wheat show a strong diurnal pattern with a marked minimum during the day (Jiang et al., 2004).

At the same time, it has to be considered that inside the NMR plant sensor part of the ear was shaded (Figure 1c). The ear and especially the glumes are photosynthetically active (Simkin et al., 2020) and partial shading may also have reduced the amount of photosynthates sequestered during the day. For this reason, in future NMR plant sensors, the rf coil and probe holder assembly should either let light in or be fitted with a light source.

A second surprising result was the rapid change in spike WC that occurred upon light on/light off, and the oscillations that followed after light on. The rapid response of PD_liq_ to light on-off events remained through seed development until day 19, which was also the last day that PD_liq_ exhibited its oscillatory behavior. These observations suggest that the xylem connectivity of kernels to the vegetative part of the plant remained intact at least until day 19. The only comparable data on the water status of monocots to our knowledge pertains to changes in leaf elongation rate in maize. It was found to exhibit a notable jump and subsequent oscillation upon sudden light off (Poire et al., 2010). Oscillations associated with water transport and plant water status have been reported in various dicots and are often termed stomatal oscillation, though the actual cause remains poorly understood (López-Bernal et al., 2018).

Barlow et al. (1980) showed that wheat grain water potential was insensitive to changes in water potential of the plant, from 10 days after anthesis onwards. On the basis of this finding it was concluded that the xylem connectivity must have been interrupted. A more recent study showed that xylem connectivity between seed and plant remained intact until much later stages of seed development (Neghliz et al., 2016). Here it was concluded that xylem occlusion may mark the end of seed filling and cause the start of the dehydration phase. Our results agree with the latter study: from day 19 onward dry matter deposition was halted, and the loss of water from the spike strongly increased.

## Conclusion

NMR plant sensors, in combination with straightforward NMR and data processing methods, provide a means to continuously, quantitatively and non-invasively monitor changes in water and dry matter content in plants. Measurements can be done with high temporal resolution (<10 min), *in vivo* and *in situ*, and for extended periods of time.

The NMR sensor setup presented here, comprising a temperature controlled small scale permanent NMR magnet and a spectrometer, can easily be moved for applications in the lab, greenhouse or field. Inside the current sensor the sample is shaded and exposed to an elevated temperature. In future versions of the sensor, the latter drawback may be addressed by using inherently temperature stable NMR magnets that do not require temperature control. Undesired shading of the sample can be prevented by opening up the probe housing or providing built-in illumination.

The ability to measure changes in water weight of intact growing leaves was demonstrated in a rice osmotic challenge experiment. The sensitivity of the method allowed to measure changes in leaf WW during the diurnal cycle of unstressed plants as well as during PEG induced osmotic challenges. Calibrated against p-v data of the same organ, changes in WW_leaf_ may also be used to monitor leaf and stem xylem water potential of the intact plant, in absolute or relative terms (Ψ, ΔΨ).

The utility of NMR sensing to measure dry matter accumulation and water content was demonstrated by continuously monitoring seed filling in wheat. Both metrics could be recorded continuously, or quantified as day and night time relative accumulation rates. Interestingly, from 5 days after booting onwards, most dry matter was deposited during the night.

## Data Availability Statement

The raw data supporting the conclusions of this article will be made available by the authors, without undue reservation.

## Author Contributions

CW conceived the measurement principle, designed and built the NMR sensor magnet, designed the experiments, did most data analysis, wrote the manuscript with contributions of all authors, and agreed to serve as the author responsible for contact and ensures communication. MN tested NMR sensor prototypes and performed and analyzed all rice experiments. JK was responsible for the design, construction, and programming of all custom built electronics hardware (rf tuning assembly, temperature control units, spectrometer housing). SJ and US supervised the project.

## Conflict of Interest

The authors declare that the research was conducted in the absence of any commercial or financial relationships that could be construed as a potential conflict of interest.
