# Supplementary material for: A Mobile NMR Sensor and Relaxometric Method to Non-destructively Monitor Water and Dry Matter Content in Plants
Source: Front Plant Sci. 2021 Feb 5;12:617768. doi: 10.3389/fpls.2021.617768 (PMC7892787; doi:10.3389/fpls.2021.617768)
Supplement: Supplementary file 1 [file Data_Sheet_1.docx]

Supplementary Material

**Supplementary Figure S1.** FID and CPMG signal amplitude scale linearly with amount of water in the sensitive volume of the NMR coil and were not affected by the increase in coil load. A test tube in the center the coil was incrementally filled a dilute CuSO_4_ reference solution with a T_2_ of 200ms (100µl steps).

**Supplementary Figure S2.** FID and CPMG signal amplitude scale linearly with amount of water in the NMR coil and were not affected by sample position. In the coil long capillaries (much longer than the rf coil) were placed, randomly distributed radially. The capillaries were filled with the same reference liquid as in Supplementary Figure S1.
